# Retrospective Comparison of Efficacy and Safety of Rabbit Anti-Thymocyte Globulin and Porcine Anti-Lymphocyte Globulin in Patients With Acquired Aplastic Anemia Undergoing Hematopoietic Stem Cell Transplantation From Matched Sibling Donors

**DOI:** 10.3389/fimmu.2022.889784

**Published:** 2022-06-01

**Authors:** Yuanfeng Zhang, Xin Chen, Lin Li, Yun Li, Li Lin, Yang Cao, Na Wang, Donglin Yang, Aiming Pang, Rongli Zhang, Qiaoling Ma, Weihua Zhai, Yi He, Jialin Wei, Erlie Jiang, MingZhe Han, Yicheng Zhang, Sizhou Feng

**Affiliations:** ^1^ State Key Laboratory of Experimental Hematology, National Clinical Research Center for Blood Diseases, Haihe Laboratory of Cell Ecosystem, Institute of Hematology and Blood Diseases Hospital, Chinese Academy of Medical Sciences and Peking Union Medical College, Tianjin, China; ^2^ Department of Hematology, Tongji Hospital, Tongji Medical College, Huazhong University of Science and Technology, Wuhan, China; ^3^ Department of Hematology, The Affiliated Yantai Yuhuangding Hospital of Qingdao University, Yantai, China; ^4^ Bone Marrow Transplantation Center, The First Affiliated Hospital, School of Medicine, Zhejiang University, Hangzhou, China

**Keywords:** rabbit, porcine, aplastic anemia, stem cell transplantation, matched sibling donors

## Abstract

We compared the efficacy and safety of porcine anti-lymphocyte globulin (pALG) (n=140) and rabbit anti-thymocyte globulin (rATG) (n=86) in patients with acquired aplastic anemia (AA) receiving hematopoietic stem cell transplantation (HSCT) from matched sibling donors (MSD) in two transplantation centers in China ranging from 2005 to 2020. The groups had similar baseline characteristics except for a higher number of infused mononuclear cells (*P*<0.001) and a higher proportion of peripheral blood stem cells as graft sources (*P*=0.003) in the pALG group. The rates of neutrophil engraftment at day 28 (*P*=1), platelet engraftment at day 28 (*P*=0.228), bloodstream infection before engraftment (*P*=0.867), invasive fungal diseases (*P*=0.362), cytomegalovirus viremia (*P*=0.667), and graft rejection (*P*=0.147) were similar in the two groups. A higher cumulative incidence of grades II-IV acute graft versus host disease (aGvHD) at 100 days occurred in the pALG group (19% vs. 8%, *P*=0.035) while no significant differences in grades III-IV aGvHD (*P*=0.572), mild to severe chronic GvHD (cGvHD) (*P*=0.181), and moderate to severe cGvHD (*P*=0.586) were observed. The actuarial 5-year overall survival (OS), failure-free survival (FFS), and GvHD-free, FFS rates of the pALG group were 87% (95% confidence interval [CI], 82-93), 85% (95% CI, 80-92), and 78% (95% CI, 72-92) versus 91% (95% CI, 86-99) (*P*=0.33), 88% (95% CI, 82-97) (*P*=0.428), and 79% (95% CI, 72-90) (*P*=0.824) in the rATG group, respectively. A busulfan-containing conditioning regimen was the only adverse risk factor for OS and FFS in multivariate analysis. In conclusion, pALG is an alternative to rATG in patients with severe AA receiving MSD-HSCT. A prospective, large-sample study is needed to explore this therapy further.

## Introduction

Severe aplastic anemia (SAA) is a disease with a high mortality rate, mainly due to infections or bleeding caused by persistent pancytopenia. Hematopoietic stem cell transplantation (HSCT) is preferred for patients with matched sibling donors (MSD). Many studies have shown significant superiority of MSD-HSCT over immunosuppressive therapy (IST) in terms of overall survival (OS) and failure-free survival (FFS) (1–5). Over the last two decades, further dramatic progress has been made on several fronts to tackle this disease. The incorporation of anti-thymocyte globulin (ATG) into the conditioning regimen was first investigated. Since 1994, the efficacy of ATG in the conditioning regimen of HSCT from MSD for patients with SAA has been confirmed (6). Storb et al. showed that the actual survival rate at three years was 92%, which was higher than the 72% (historical) survival rate, in 39 patients. In addition, a fludarabine (FLU)-based conditioning regimen also showed reduced toxicity and similar survival compared to ATG plus cyclophosphamide (CTX), especially for older patients (7–9). However, among these studies, rabbit-ATG (rATG) was most commonly used. In China, porcine anti-lymphocyte globulin (pALG) is also available, and studies have reported similar efficacy to rATG among patients receiving IST (10–13). The effect and safety of pALG in patients with SAA receiving HSCT have been previously reported in our centers, with limited sample sizes (14, 15). Therefore, we designed this extended retrospective study to evaluate and compare the efficacy and safety of rATG and pALG in patients with acquired SAA undergoing MSD-HSCT in two transplant centers in China.

## Patients and Methods

### Patients

From 2005 and 2020, a total of 226 patients with acquired AA who consecutively received MSD-HSCT from Institute of Hematology & Blood Diseases Hospital, Chinese Academy of Medical Sciences & Peking Union Medical College and Tongji Hospital, Tongji Medical College, Huazhong University of Science and Technology were enrolled in this study. Of these patients evaluated, 140 patients received pALG while 86 patients received rATG. Enrollment criteria included SAA, very SAA or transfusion-dependent non-severe AA defined by guideline (16); voluntary participation in HSCT; absence of severe organs dysfunction. Excluding criteria included underlying inherited marrow failure disorders such as Fanconi anemia; myelodysplastic syndrome; patients with pregnancy, severe organs impairment, or uncontrolled active infection. Patients with paroxysmal nocturnal hemoglobinuria clones were also included in this study. All written informed consent was attained from patients or their relatives. This study was approved by the Ethics Committees of Institute of Hematology & Blood Diseases Hospital, Chinese Academy of Medical Sciences & Peking Union Medical College and Tongji Hospital, Tongji Medical College, Huazhong University of Science and Technology, respectively.

### Procedures of Transplantation

The conditioning regimen, graft-versus-host disease (GvHD) prophylaxis, infection prevention, and surveillance followed those in our previous report (14, 15). In particular, pALG was prepared using thymic cells (as antigens) introduced in swine and separating anti-lymphocyte serum from the swine (Wuhan Institute of Biological Products Co., Ltd.) (11, 17).

### Definitions

Neutrophil and platelet engraftment (18), acute GvHD (aGvHD) (19), and chronic GvHD (cGvHD) (20) were defined according to previously reported criteria. Graft rejection (GR) was defined as less than 5% T-cells of donor origin (21). Primary GR was defined as the failure to achieve neutrophil engraftment after HSCT until day +28. Secondary GR was defined as the absence of graft function after achieving initial full engraftment (22). Transplantation-related mortality (TRM) was defined as death without rejection. Treatment failure after HSCT was defined as death or primary or secondary GR, whichever came first. The FFS was defined as survival without treatment failure. GvHD-free and failure-free survival (GFFS) was defined as survival without grades III-IV aGvHD, moderate to severe cGvHD, or treatment failure (23). OS was defined as the time from treatment to death or the last follow-up.

### Statistical Analysis

The objective of this study was to compare major outcomes, including engraftment, infection, GvHD, TRM, and survival, among different ATG groups in patients with AA.

Each patient involved had an electronic-database, outpatient-department, or telephone follow-up. The final follow-up was November 31, 2021. Continuous and categorical variables were compared using the Mann–Whitney U test, chi-square test, or Fisher’s exact test. Median follow-up was calculated using the reverse Kaplan–Meier method. Cumulative incidences of GvHD were compared with the Gray’s test. Death and GR were considered as competing events for GvHD. The probabilities of OS and FFS were estimated using the Kaplan–Meier method and compared between different groups of patients using the log-rank test. Variables with *P* values ≤ 0.2 in the univariate analysis were entered in multivariate analyses using a Cox proportional hazards model to identify factors impacting OS, FFS, and GFFS of transplant patients. Statistical analyses were performed using the R software packages (R 4.1.2), GraphPad Prism 5, and SPSS 20.0. GraphPad Prism 5 was used to generate figures. All *P* values were two-sided, and the results were considered statistically significant at *P*<0.05.

## Results

### Characteristic of Patients and Donors

As shown in **Table 1**, 140 and 86 patients were enrolled in the pALG and rATG groups, respectively. There were no significant differences in terms of patient age (*P*=0.15), patient sex (*P*=0.857), donor sex (*P*=0.797), diagnosis (*P*=0.396), interval from diagnosis to transplantation (*P*=0.375), conditioning regimen (*P*=0.415), and dose of CD34^+^ cells infused (*P*=0.161) between the two groups, while the pALG group had a higher proportion of peripheral blood stem cells (PBSCs) as a graft source (85% vs. 67.44%, *P*=0.003), and a higher median dose of infused mononuclear cells (10×10^8^/kg vs. 8×10^8^/kg, *P*<0.001).

**Table 1:** Characteristics and outcomes of patients with acquired aplastic anemia.

Variables	pALG group (140)	rATG group (86)	*P* value
Patient age, years, median (range)	26 (7-66)	24 (4-54)	0.15
Patient gender (male), no. (%)	80 (57.14)	51 (59.30)	0.857
Donor gender (male), no. (%)	64 (45.71)	37 (43.02)	0.797
Diagnosis, no. (%)			0.396
severe aplastic anemia	90 (64.29)	49 (56.98)	
very severe aplastic anemia	40 (28.57)	32 (37.21)	
non-severe aplastic anemia	10 (7.14)	5 (5.81)	
Interval from diagnosis to transplant, moths, median (range)	2 (0.4-204)	2 (0.5-231)	0.375
Conditioning regimen			0.415
ATG+CTX ± FLU	115 (82.14)	66 (76.74)	
BU+FLU+ATG ± CTX	25 (17.86)	20 (23.26)	
Graft source, no. (%)			0.003
Peripheral blood	119 (85.00)	58 (67.44)	
Bone marrow ± peripheral blood	21 (15.00)	28 (32.56)	
Mononuclear cells, ×10^8^/kg, median (range)	10 (2.8-47)	8 (2.8-38)	<0.001
CD3^+^ cells, ×10^6^/kg, median (range)	127.3 (13.7-384.1)**^&^**	115.8 (6.7-292.5)**^&^**	0.377
CD34^+^ cells, ×10^6^/kg, median (range)	3 (1.5-17)	3 (0.75-10)	0.161
Neutrophil engraftment, days, median (range)	12 (7-22)	12 (9-23)	0.004
Platelet engraftment, days, median (range)	12 (7-30)	14 (8-34)	0.001
28-day neutrophil engraftment, no. (%)	138 (100)	86 (100)	1
28-day platelet engraftment, no. (%)	132 (95.65)	78 (90.70)	0.228
Graft rejection			0.147
Primary graft rejection, no. (%)	1 (0.71)	0 (0)	
Secondary graft rejection, no. (%)	3 (2.14)	6 (6.98)	
Bloodstream infection before engraftment, no. (%)	22 (15.71)	12 (13.95)	0.867
Invasive fungal diseases, no. (%)	10 (7.14)	10 (11.63)	0.362
Cytomegalovirus viremia, no. (%)	31 (22.14)	22 (25.58)	0.667
100-day aGvHD grades I-IV, no. (%)	33 (24.26)^*^	20 (23.26)^*^	0.992
100-day aGvHD grades II-IV, no. (%)	26 (19.12)^*^	7 (8.14)^*^	0.041
100-day aGvHD grades III-IV, no. (%)	11 (8.09)^*^	5 (5.81)^*^	0.71
Mild to severe cGvHD, no. (%)	29 (22.31)^#^	9 (11.11)^#^	0.061
Moderate to severe cGvHD, no. (%)	6 (4.76)^#^	6 (7.69)^#^	0.577
Overall deaths, no. (%)	19 (13.57)	8 (9.30)	0.454
Follow-up of alive patients, moths, median (range)	62 (7-190)	79 (3-169)	0.087

*Among enrolled patients, 136 and 86 patients were evaluable; ^#^among enrolled patients, 130 and 81 patients were evaluable; ^**&**^among enrolled patients, 74 and 33 patients were evaluable.

pALG, porcine anti-lymphocyte globulin; rATG, rabbit anti-thymocyte globulin; no., number of patients; CTX, cyclophosphamide; FLU, fludarabine; BU, busulfan; aGvHD, acute graft versus host disease; cGvHD, chronic graft versus host disease.

### Hematopoietic Recovery

Only patients who survived for >28 days were analyzed for engraftment. There were two early deaths due to respiratory failure and septic shock at day 11 and 15 in the pALG group, while none of the patients in the rATG group suffered early deaths. The neutrophil engraftment rate was 100% at day 28 in the pALG group versus 100% at day 28 in the rATG group; accordingly, the platelet engraftment rate was 96.65% versus 90.7%, respectively (*P*=0.228). Patients in the pALG group had a faster engraftment of neutrophils and platelets. The median days of neutrophil and platelet engraftment were 12 (range, 7-22) and 12 (range, 7-30) days for patients in the pALG group and 12 (range, 9-23) (*P*=0.004) and 14 (range, 8-34) days (*P*=0.001) for patients in the rATG group, respectively (**Table 1**).

### Graft Rejection

Ten patients experienced GR after transplantation (one primary and nine secondary). There was no difference in GR rates between the groups (*P*=0.147). The median time of secondary GR was 4 months, ranging from 1.2 months to 107 months. Among these patients, all were treated with CTX (50 mg/kg × 2 days) with or without FLU followed by an infusion of frozen PBSCs from the original donor. Eight patients acquired complete blood recovery with donor origin, while two patients received autologous blood recovery.

### aGvHD and cGvHD

**Figure 1:**
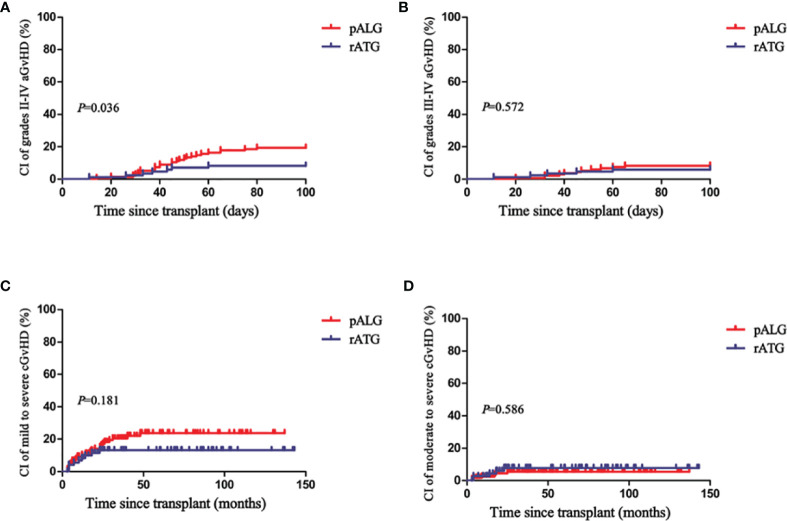
Cumulative incidences (CI) of grades II-IV aGvHD **(A)**, grades III-IV aGvHD **(B)**, mild to severe cGvHD **(C)**, and moderate to severe cGvHD **(D)** in the pALG and rATG groups.

With regard to aGvHD and cGvHD, although patients in the pALG group mostly received PBSCs as the graft source, there was only a marginally significant difference in the rate of grades II to IV aGvHD between the two groups (*P*=0.041), whereas rates of grades I to IV aGvHD, grades III to IV aGvHD, mild to severe cGvHD, and moderate to severe cGvHD were similar (**Table 1**). **Figure 1** shows that the cumulative incidences of grades II to IV aGvHD and III to IV aGvHD at 100 days were 19% (95% confidence interval [CI], 6–30) and 8% (95% CI, 0–15) in the pALG group compared to 8% (95% CI, 0–16) (*P*=0.035) and 6% (95% CI, 0–12) (*P*=0.572), respectively, in the rATG group. The cumulative incidence of mild-to-severe and moderate-to-severe cGvHD at 5 years was 24% (95% CI, 9–36) and 5% (95% CI, 0–11) in the pALG group versus 13% (95% CI, 0–25) (*P*=0.181) and 8% (95% CI, 0–16) (*P*=0.586) in the rATG group.

### Infections

There were no statistical differences in bloodstream infections before engraftment (*P*=0.867), invasive fungal diseases (*P*=0.362), or cytomegalovirus viremia (*P*=0.667) between the two groups (**Table 1**).

### Deaths

With a median follow-up of 62 months (range, 7-190 months) and 79 months (range, 3-169 months), 19 and 8 deaths occurred in the pALG and rATG groups (*P*=0.454), respectively. The primary causes of death (COD) are listed in **Table 2**. The leading COD was infection (n=13), followed by aGvHD (n=8). Remarkably, four patients died from invasive fungal diseases of the lung (n=3) or brain (n=1) before 2010. Secondary COD followed by aGvHD included infections (n=4), organ failure (n=3), and gastrointestinal bleeding (n=1). More patients receiving busulfan (BU)-containing regimens suffered COD owing to aGvHD (11.1% vs. 1.7%, *P*=0.009), whereas COD caused by infection between the two conditioning groups was similar (6.1% vs. 2.2%, *P*=0.468).

**Table 2:** Primary causes of death (COD) among patients.

COD	pALG group (n=19) (%)	rATG group (n=8) (%)	*P* value
aGvHD	7 (5)	1 (1.2)	0.16
Infection	10 (7.1)	3 (3.5)	0.379
cGvHD	1 (0.7)	–	1
Accident	1 (0.7)	1 (1.2)	1
Intracranial hemorrhage	–	3 (3.5)	0.054

pALG, porcine anti-lymphocyte globulin; rATG, rabbit anti-thymocyte globulin; aGvHD, acute graft versus host disease; cGvHD, chronic graft versus host disease.

### Survival

**Figure 2:**
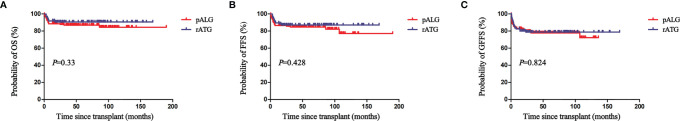
The actuarial 5-year overall survival (OS) **(A)**, failure-free survival (FFS) **(B)**, and GvHD-free, failure-free survival (GFFS) **(C)** rates of patients in the pALG and rATG groups.

**Figure 3:**
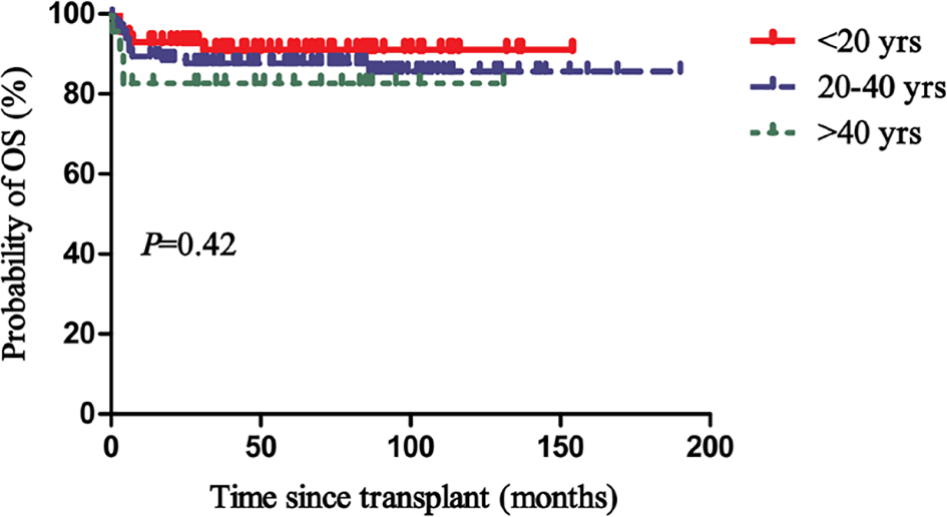
Overall survival (OS) of different age groups.

The actuarial 5-year OS, FFS, and GFFS rates of the pALG group were 87% (95% CI, 82-93), 85% (95% CI, 80-92), and 78% (95% CI, 72-92) compared to 91% (95% CI, 86-99) (*P*=0.33), 88% (95% CI, 82-97) (*P*=0.428), and 79% (95% CI, 72-90) (*P*=0.824) of the rATG group, respectively (**Figure 2**). In the subgroup analysis, the actuarial 5-year OS rates of patients aged<20 years, 20-40 years, and >40 years were 91% (95% CI, 86-100), 88% (95% CI, 83-95), and 83% (95% CI, 72-100) (*P*=0.42) (**Figure 3**), respectively.

As shown in **Table 3**, in univariate and multivariate analysis, a BU-containing regimen was the only adverse risk factor of OS and FFS.

**Table 3:** Univariate and multivariate analysis of survival.

Variables	Comparison	Overall survival				Failure-free survival			
		Univariate analysis		Multivariate analysis		Univariate analysis		Multivariate analysis	
		HR (95%CI)	*P* value	HR (95%CI)	*P* value	HR (95%CI)	*P* value	HR (95%CI)	*P* value
Patient gender	Female vs male	0.44 (0.19-1.05)	0.065	0.47 (0.17-1.29)	0.144	0.53 (0.25-1.1)	0.088	0.96 (0.3-1.32)	0.221
Donor gender	Female vs male	1.42 (0.65-3.11)	0.377			1.19 (0.6-2.35)	0.62		
Patient age	Continuous variable	1.02 (0.99-1.05)	0.269			1.01 (0.98-1.04)	0.503		
Diagnosis (VSAA)	VSAA vs SAA	1.71 (0.77-3.82)	0.19	2.05 (0.81-5.14)	0.128	1.84 (0.89-3.77)	0.097	1.94 (0.92-4.07)	0.082
Diagnosis (NSAA)	NSAA vs SAA	2.13 (0.61-7.47)	0.238	1 (0.21-4.77)	1	2.45 (0.82-7.34)	0.109	1.74 (0.56-5.4)	0.337
Treatment	ATG vs no ATG	1.45 (0.2-10.73)	0.715			1.14 (0.16-8.36)	0.898		
ATG source	rATG vs pALG	0.67 (0.29-1.52)	0.336			0.75 (0.36-1.54)	0.43		
Conditioning regimen	Bu vs non-Bu	3 (1.39-6.49)	0.005	3.4 (1.35-8.56)	0.009	2.07 (1.01-4.27)	0.048	0.35 (0.1-1.15)	0.031
Interval from D to T	Continuous variable	1 (0.99-1.01)	0.395			1 (0.99-1.01)	0.617		
Graft source	BM ± PB vs PB	0.43 (0.13-1.42)	0.167	0.65 (0.15-2.86)	0.566	0.31 (0.1-1.03)	0.056	2.35 (1.08-5.1)	0.084
Amount of MNC	Continuous variable	0.99 (0.93-1.05)	0.66			0.98 (0.93-1.04)	0.462		
Amount of CD34^+^ cells	Continuous variable	0.99 (0.84-1.16)	0.871			0.97 (0.83-1.12)	0.651		

HR, hazard ratio; CI, confidence interval; VSAA, very severe aplastic anemia; NSAA, non-severe aplastic anemia; SAA, severe aplastic anemia; ATG, anti-thymocyte globulin; rATG, rabbit ATG; pALG, porcine anti-lymphocyte globulin; D, diagnosis; T, transplantation; Bu, busulfan; BM, bone marrow; PB, peripheral blood; MNC, mononuclear cells.

## Discussion and Conclusion

MSD-HSCT, which promotes effective and fast recovery of blood counts, is the preferred treatment for young patients with SAA. As SAA is a nonmalignant disease, it is recommended to sustain engraftment and minimize GvHD by modifying the conditioning regimen. High-dose CTX plus ATG is the standard conditioning regimen for SAA patients undergoing MSD-HSCT (24, 25). The addition of ATG to CTX reduces GR and GvHD rates (6). In different multivariate analyses, a conditioning regimen without ATG was a negative risk factor for survival in patients with SAA who received HSCT (26–28).

However, the mechanisms of ATG in conditioning are not well understood. It plays a role in suppressing recipient T cells to promote engraftment, as well as donor-activating T cells to reduce GvHD (29). There are three types of ATG worldwide. *In vivo* studies have demonstrated that the immunosuppressive effect of rATG was stronger than that of horse ATG (hATG) in SAA (30, 31); on the other hand, more infections and lower rates of aGvHD were related to rATG for patients with SAA receiving HSCT (32, 33). In China, no hATG or pALG has been approved by the China Food and Drug Administration as a drug in the conditioning regimen for transplantation. Several studies have demonstrated comparable outcomes between pALG and rATG as IST in patients with SAA (10–13). Previously, another study in IST has demonstrated that compared to pALG, r-ATG exhibited a stronger and prolonged inhibition effect on the CD4^+^ T cell subset while a subset of CD4^+^ T cells played a role in hematopoietic recovery (12).

Consistent with our previous study (14), we found no differences in the rates of neutrophil engraftment (*P*=1), platelet engraftment (*P*=0.228), bloodstream infections (*P*=0.867), invasive fungal diseases (*P*=0.362), cytomegalovirus viremia (*P*=0.667), or GR (*P*=0.147) between the two groups. Patients in the pALG group experienced faster recovery of neutrophils (*P*=0.004) and platelet (*P*=0.001). Meanwhile, a higher cumulative incidence of grades II-IV aGvHD at 100 days occurred in the pALG group (19% vs. 8%, *P*=0.036), while no differences were observed in the cumulative incidence of grades III-IV aGvHD (*P*=0.572), mild to severe cGvHD (*P*=0.181), and moderate to severe cGvHD (*P*=0.586). Schrezenmeier et al. reported the median days of neutrophil and platelet engraftment were 13 and 19 days in 134 PB recipients compared to 19 and 25 days in 558 bone marrow (BM)recipients of MSD-HSCT for SAA (34). Bacigalupo et al. have compared the efficacy of PB (n=723) with BM (n=1138) as graft sources for patients with AA receiving MSD-HSCT. They demonstrated that the median days of neutrophil and platelet engraftment in PB patients were 15 (5-68) and 15 (5-68) days versus 20 (3-156) and 27 (4-305) days in BM patients. Grades II to IV aGvHD of PB patients was higher than that of BM patients (17% vs. 11%, *P*=0.001) (26). Therefore, we should notice that a higher proportion of PB (*P*=0.003) as a graft source and a higher amount of infused MNC (*P*<0.001) in the pALG group may lead to faster recovery in the WBC and PLT engraftment as well as a higher rate of grades II to IV aGvHD. Even so, the actuarial 5-year OS, FFS, and GFFS rates between our two groups were similar.

In our study, we applied a non-myeloablative conditioning regimen consisting of FLU, a reduced dose of CTX, and ATG in patients with acquired AA. Several studies have reported similar efficacy of FLU-based conditioning regimens for SAA compared with a standard dose of CTX plus ATG conditioning regimen, especially for patients older than 30 years (7–9). Usually, a dose of BU 6.4 mg/kg was added to patients with a high risk of graft failure, for instance, patients with long intervals from diagnosis to transplantation or heavy blood cell transfusion. Based on the intensity of conditioning (35), this is defined as reduced-intensity conditioning. Only one patient in our study experienced primary GR. Although we demonstrated that a BU-containing conditioning regimen was an adverse predictor of OS, and FFS, these results should be interpreted with critical caution. As we know, the interval from diagnosis to transplantation and heavy transfusions before transplantation are associated with poor outcomes in patients with SAA, which may impact these results as well (26, 27, 36). Meanwhile, enhancing the intensity of the conditioning regimen may improve engraftment at the cost of more toxicity, as revealed by a meta-analysis (37). In our study, we found that more patients receiving a BU-containing conditioning regimen died of aGvHD (*P*=0.009). Notably, none of our patients with GR died, and most of them were successfully salvaged by the original donors’ PBSC infusion. In the subgroup analysis, there was no difference in OS among patient age groups, which indicated that this regimen may be applied to older patients (8). Therefore, these results indicate that a fludarabine-based conditioning regimen was effective for patients with SAA undergoing MSD-HSCT, independent of age.

Our study had several limitations. First, it was a retrospective study with unavoidable bias. Notably, our enrolled patients had relatively similar basic characteristics to minimize the effect of potential bias. Second, our data on the rates of full immune reconstitution at different times between the two groups was incomplete. In the future, we could use this as a useful secondary endpoint in prospective studies. Third, longer follow-up is necessary as the significant difference in cGvHD rate after PB and BM allografts was most obvious with follow-ups of more than 6 to 7 years (34).

In summary, our study showed that pALG is an alternative treatment for patients with SAA undergoing HSCT from an MSD. Its safety and efficacy were similar to those of rATG. A prospective, large-sample study is needed to validate our findings.

## Data Availability Statement

The original contributions presented in the study are included in the article/supplementary material. Further inquiries can be directed to the corresponding authors.

## Ethics Statement

The studies involving human participants were reviewed and approved by the Ethics Committees of Institute of Hematology & Blood Diseases Hospital, Chinese Academy of Medical Sciences & Peking Union Medical College and Tongji Hospital, Tongji Medical College, Huazhong University of Science and Technology. Written informed consent to participate in this study was provided by the participants’ legal guardian/next of kin.

## Author Contributions

SF and YcZ contributed to the study design and manuscript review. YfZ, XC, LinL, YL, LiL, GY, and YN contributed to data collection and analysis. YfZ wrote the manuscript, and YfZ, XC, and LinL performed statistical analyses. XC, AP, DY, RZ, QM, WZ, YH, JW, EJ, and MH contributed to disease treatment and data collection. All authors have contributed to the manuscript and approved the submitted version.

## Funding

The authors disclose receipt of the following financial support for the research, authorship, and/or publication of this article: This work was supported by the CAMS Innovation Fund for Medical Sciences (CIFMS) (grant numbers 2021-1-I2M-017 and 2021-I2M-C&T-B-080) and the Youth Program of the National Natural Science Foundation of China (grant number 81900182).

## Conflict of Interest

The authors declare that the research was conducted in the absence of any commercial or financial relationships that could be construed as a potential conflict of interest.

## Publisher’s Note

All claims expressed in this article are solely those of the authors and do not necessarily represent those of their affiliated organizations, or those of the publisher, the editors and the reviewers. Any product that may be evaluated in this article, or claim that may be made by its manufacturer, is not guaranteed or endorsed by the publisher.
